# Development and internal verification of nomogram for forecasting delirium in the elderly admitted to intensive care units: an analysis of MIMIC-IV database

**DOI:** 10.3389/fneur.2025.1580125

**Published:** 2025-05-13

**Authors:** Li Jiang, Dongdong Yu, Ge Yang, Xiaoqian Wu, Dong Zhang

**Affiliations:** Department of Anesthesiology, Hebei General Hospital, Shijiazhuang, Hebei, China

**Keywords:** delirium, prediction model, nomogram, elderly, intensive care unit

## Abstract

**Background:**

Precise forecasting of delirium in intensive care unit (ICU) may propel effective early prevention strategies and stratification of ICU patients through delirium risks, avoiding waste of medical resources. However, there are few optimal models of delirium in critically ill older patients. This study aimed to propose and verify a nomogram for predicting the incidence of delirium in elderly patients admitted to ICU.

**Methods:**

We performed a retrospective study using data from the Medical Information Mart for Intensive Care IV (MIMIC-IV) database. It included data on 13,175 older patients in total. The patients were randomly divided into a training group (*n* = 9,223) and an internal verification group (*n* = 3,452). Risk factors were screened using the least absolute shrinkage and selection operator regression. We successfully constructed a multivariate logistic regression model along with a nomogram. We conducted internal verification using 1,000 bootstrap specimens. Performance assessment was conducted using a receiver operating characteristic (ROC) curve, calibration curve, decision curve analysis (DCA), and clinical impact curve (CIC).

**Results:**

The risk factors included in the nomogram were sepsis, Sequential Organ Failure Assessment (SOFA) score, cerebrovascular disease, mechanical ventilation, sedation, severe hypothermia, and serum calcium levels. The area under the ROC curve (AUC) for the nomogram, incorporating the above-mentioned predictors for the training set was 0.762 (95% confidence interval [CI] 0.749–0.776), whereas that for the verification set was 0.756 (95% CI 0.736–0.776). Based on the calibration curve, the model forecast outcomes matched well with the actual results, and the nomogram’s Brier score was 0.12 in the training set and 0.128 in the verification set. DCA and CIC showed that our model had a good net clinical benefit.

**Conclusion:**

We developed a forecast nomogram for delirium in the critically ill elderly patients that enhances clinical decision-making. However, further verification is required.

## Introduction

In view of the progressive degradation of physiological and cognitive functions, elderly patients are vulnerable to various complications following intensive care unit (ICU) admission, of which delirium is one of the most frequent (1). Delirium is characterized by inattention, acute fluctuations or variations in cognitive functions, confusion, and changing consciousness. It is considered as one of the most prevalent neuropsychological complications in elderly patients during their ICU stay. According to previous reports, ICU delirium incidence rates in elderly patients are between 24 and 66%, depending on the screening instrument and patient population (2–4). This leads to increased morbidity and mortality, increased admission rates to long-time care facilities, prolonged hospital stays, increased postoperative complications, and poorer functional outcomes (5, 6). Given these adverse consequences, prevention of delirium is essential.

Many studies have confirmed that the occurrence of delirium may be significantly decreased by timely preventive interventions aimed at various delirium risk factors (7, 8). However, it has been documented that non-pharmacological approaches are effective in preventing delirium, whereas controversy remains regarding whether medications can prevent and treat delirium (9). Therefore, non-pharmacological interventions remain the foundation for the treatment of delirium. Notably, bundle administration strategies for critically ill patients at risk of delirium have attracted increasing attention. The ABCDEF bundle (Assess, prevent, and manage pain; Both spontaneous awakening and spontaneous breathing trials; Choice of analgesia and sedation; Delirium: assess, prevent, and manage; Early mobility and exercise; and Family engagement and empowerment), which is recommended by the Society of Critical Care Medicine (SCCM) can considerably enhance the prevention of delirium (10). However, due to workload and documentation burden, overall bundle adherence is poor in clinical practice with implementation rates of 12–57% (11–13).

In the case of limited resources, a precise and functional assessment of delirium risk in ICU patients is the basis for efficient and effective interventions. A reliable forecast model for delirium will help clinical therapists identify patients at high delirium risk and provide them with timely interventions. Machine learning models have shown effectiveness in predicting delirium, however, the lack of transparency in prediction processes reduced the interpretability and limited their utility in guiding clinical interventions. Additionally, the complexity of model development and the requirement for large datasets hinder their widespread clinical application. Nomograms, as simpler predictive tools, allow for intuitive visualization of variable contributions to outcomes, offering better interpretability and clinical utility. Although numerous studies have developed nomogram models for delirium prediction, the foundation for such models is either findings from patients with wide age ranges (14, 15) or from solely specific ICU units (14, 16), failing to account for generalizability to older ICU patients. This study aimed to develop and verify a predictive model for delirium in elderly ICU patients (over 65-year-old) using a large clinical database. The findings of this study will assist in the early identification of ICU patients with the highest risk of delirium, thus allowing clinical therapists to conduct timely preventive interventions.

## Materials and methods

### Study design

This retrospective cohort study was conducted using the Medical Information Mart for Intensive Care IV (MIMIC-IV) database (version 2.0.). This database includes information on ICU patients admitted to Beth Israel Deacon-ess Medical Centre in Boston, Massachusetts between 2008 and 2019 (17). One author (LJ) completed the Collaborative Institutional Training Initiative examination and obtained access to the database (certification number: 9771318). The institutional review boards of Beth Israel Deaconess Medical Centre and the Massachusetts Institute of Technology Affiliates approved the application for the MIMIC-IV database. Personal data in this database were processed; therefore, informed consent was not required. We conducted the study according to the Transparent Reporting of a Multivariable forecast Model for Individual Prognosis or Diagnosis (TRIPOD) statement (18).

### Research population and data extraction

**Figure 1:**
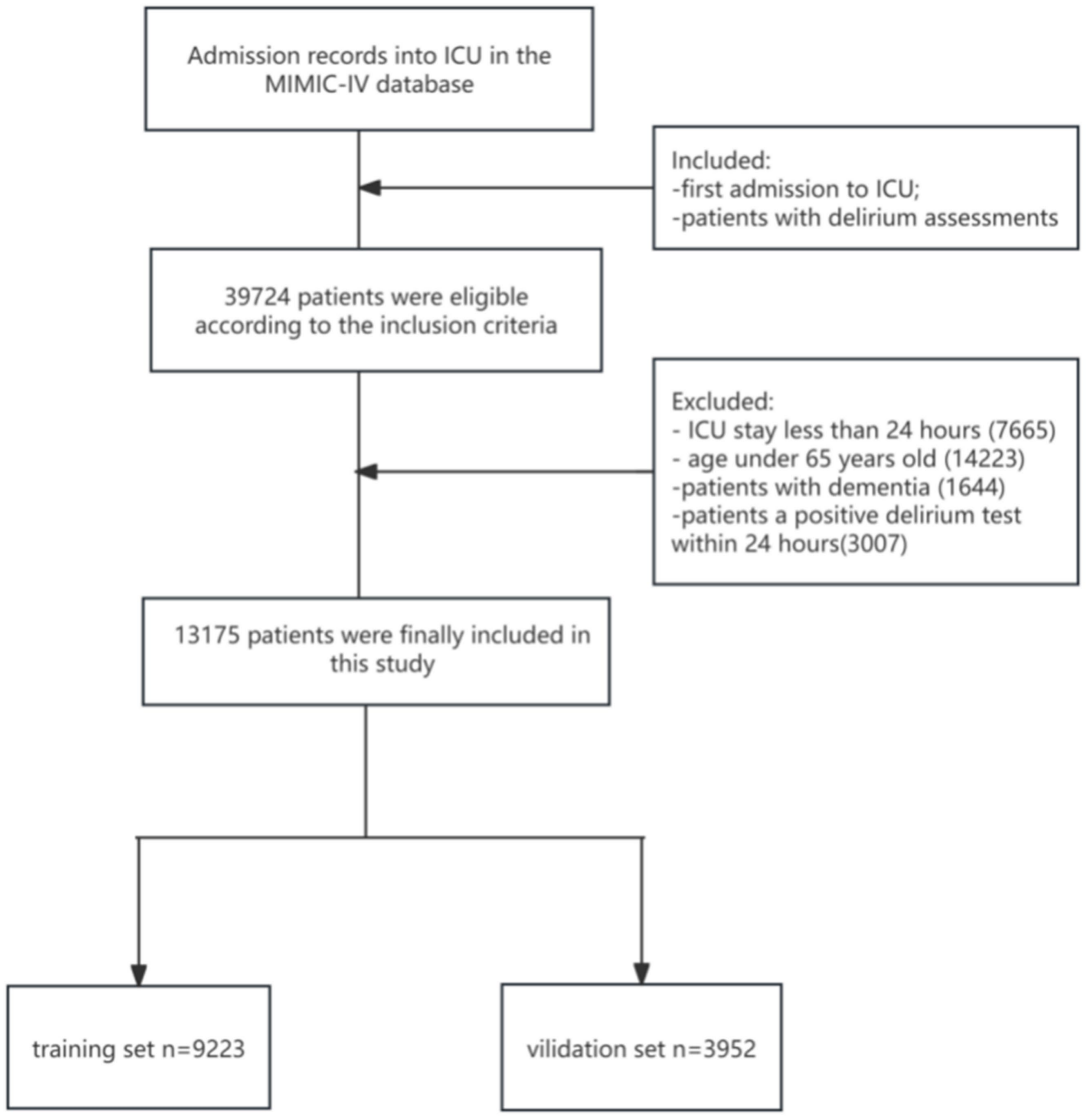
The flow diagram of enrollment procedure. ICU, intensive care unit; MIMIC-IV, Medical Information Mart for Intensive Care-IV.

Figure 1 shows the enrollment procedure of the study patients. We included patients who completed delirium evaluation during their initial ICU admission in the MIMIC-IV database. Patients who stayed in the ICU for less than 24 h or who were under 65 years were excluded from the study. Patients diagnosed with dementia were also excluded because such patients are often misdiagnosed as having cognitive impairment. To make patients less likely to develop delirium prior to ICU admission, patients with a positive delirium test within 24 h after admission were also excluded (19, 20). Therefore, our study included 13,175 patients totally.

We extracted all research data from MIMIC-IV using structured query language (SQL) with Navicat Premium. The data included (1) patient demographics; (2) first-day vital signs and laboratory indicators including respiratory rate (RR), mean blood pressure (MBP), temperature: severe hypothermia (<35.0°C), mild hypothermia (35.0°C to <36.0°C), and normothermia (≥36.0°C) (21), heart rate (HR), SpO_2_, hemoglobin, platelet, white blood cell (WBC), chloride, calcium, sodium, potassium, bicarbonate, blood urea nitrogen (BUN), creatinine, prothrombin time (PT), partial thromboplastin time (PTT), international normalized ratio (INR), and glucose; (3) relevant comorbidities and scores that reflect patient disease severity, such as cerebrovascular disease (stroke, cerebral infarction, cerebral hemorrhage, stenosis and transient ischemic attack), sepsis (organ dysfunction, hypoperfusion, or hypotension caused by systemic infection based upon the sepsis 3.0 Definition) and Sequential Organ Failure Assessment (SOFA) score; (4) mechanical ventilation (invasive ventilation only) and sedation (use lorazepam, diazepam, midazolam and propofol) within 24 h of ICU admission; and (5) details of admission. For variable data with several measurements, we included the minimum value within the first 24 h of SpO_2_ and hemoglobin levels in analysis, whereas we included the maximum values of other variables for analysis. We excluded variables with over 20% missing data and the remaining variables with missing values underwent multiple imputation using “MICE” package in R (22). See Supplementary Table S1 for detailed data.

### Delirium assessment

In the MIMIC database, delirium screening was conducted using the Confusion Assessment Method in ICU. It is a verified ICU bedside device for routine delirium monitoring with high reliability, sensitivity, and specificity (23). We defined patients scoring positive for delirium as having one or more positive delirium screenings at any ICU stay, whereas we defined those scoring negative for delirium as possessing all negative outcomes in delirium screening.

### Statistical analysis

The study population was divided into two groups according to their delirium assessment. The distribution of data was analyzed by the Shapiro–Wilk test. Continuous variables with a normal distribution were expressed as the mean ± standard deviation (SD) and were compared using the independent-sample *t*-test. The Mann–Whitney U test presented continuous variables with a non-normal distribution. Categorical data were presented as a number (%) and were analyzed using the chi-square test or Fisher’s exact probability test.

To develop the forecast nomogram, we randomly divided patients into a training or verification set in a 7:3 ratio. Variable selection and model development included all patients in the training set. Delirium predictors were screened using the least absolute shrinkage and selection operator (LASSO) expression with 95% confidence intervals (95% CIs) and odds ratios (ORs) computed (24). Variables included in the model were determined via the largest *λ* value with a mean error within a single standard deviation chosen as per cross-verification outcomes (bootstrap resampling 1,000 times). Model performance was assessed in the training and verification sets using the area under the receiver operating characteristic curve (AUC). The degree of consistency of the actual and forecast results was assessed using the calibration curve, Brier score, and Hosmer–Lemeshow goodness of fit test (HL test). The clinical application of the nomogram was assessed using a clinical impact curve (CIC) and decision curve analysis (DCA).

All statistical analyses were performed using Free Statistics software (version 2.0) and R software packages (The R Foundation)^1^ (25). Statistical significance was set at *p* < 0.05.

## Results

### Patient clinical and demographic data

Finally, our research included 13,175 older ICU patients. See Figure 1 for the elaborate selection process. The enrolled patients included 2,300 patients with delirium (17.5%). Table 1 summarizes the baseline characteristics and details within 24 h of ICU patient admission. Compared with the non-delirium group, patients with delirium were older, and their RR and HR were faster. In addition, these patients had higher creatinine, platelet, BUN, WBC, INR, PT, PTT, and glucose levels and a greater possibility of sepsis; peripheral vascular, chronic pulmonary, and cerebrovascular diseases; congestive heart failure; and renal and liver illnesses. Moreover, patients in the delirium group received more medical treatment and had significantly higher SOFA scores. Patients with delirium had a more unfavorable prognosis. Their hospital and ICU stay periods were much longer and their 30-day mortality rates were higher too.

**Table 1:** Baseline characteristics of enrollment patients.

Variables	Total (*n* = 13,175)	No-delirium (*n* = 10,875)	Delirium (*n* = 2,300)	*p*	Statistic
Age	75.8(70.2, 82.5)	75.6 (70.1, 82.4)	76.4 (70.7, 83.0)	0.01	6.548
Gender, n (%)				0.788	0.072
Female	5,821 (44.2)	4,799 (44.1)	1,022 (44.4)		
Male	7,354 (55.8)	6,076 (55.9)	1,278 (55.6)		
First care unit, n (%)				< 0.001	224.927
CVICU	3,926 (29.8)	3,437 (31.6)	489 (21.3)		
CCU	1909 (14.5)	1,641 (15.1)	268 (11.7)		
MICU	1,679 (12.7)	1,320 (12.1)	359 (15.6)		
MICU/SICU	1730 (13.1)	1,436 (13.2)	294 (12.8)		
NICU/NSICU	956 (7.3)	766 (7)	190 (8.3)		
SICU	1,672 (12.7)	1,299 (11.9)	373 (16.2)		
TSICU	1,303 (9.9)	976 (9)	327 (14.2)		
Heart rate (beats/min)	81.7 ± 14.5	81.1 ± 14.0	84.6 ± 16.1	< 0.001	114.487
MBP (mmHg)	76.9 ± 10.0	76.9 ± 10.0	76.8 ± 10.1	0.841	0.04
SpO_2_ (%)	96.8 ± 2.0	96.8 ± 1.9	96.9 ± 2.1	0.001	10.695
Resp rate (beats/min)	19.0 ± 3.5	18.9 ± 3.4	19.7 ± 3.7	< 0.001	119.027
Temperature (°C)	36.8 ± 0.4	36.8 ± 0.5	36.8 ± 0.4	< 0.001	64.724
Hemoglobin (g/dL)	10.0 ± 2.2	10.0 ± 2.1	9.9 ± 2.2	0.005	7.759
Platelets (K/μL)	213.2 ± 101.2	212.3 ± 100.4	217.4 ± 104.4	0.03	4.709
WBC (K/μL)	12.5 (9.2, 17.1)	12.4 (9.0, 16.8)	13.5 (9.9, 18.4)	< 0.001	64.994
Calcium (mmol/L)	8.6 ± 1.0	8.6 ± 0.7	8.7 ± 1.7	< 0.001	16.183
Chloride (mmol/L)	105.7 ± 5.8	105.7 ± 5.7	105.7 ± 6.3	0.987	0
Sodium (mmol/L)	139.5 ± 4.4	139.4 ± 4.3	140.0 ± 4.9	< 0.001	34.767
Potassium (mmol/L)	4.6 ± 0.8	4.6 ± 0.7	4.7 ± 0.9	< 0.001	46.294
Bicarbonate (mmol/L)	24.3 ± 3.8	24.4 ± 3.6	23.9 ± 4.3	< 0.001	27.527
Bun (mg/dL)	28.3 ± 21.4	27.3 ± 20.4	33.0 ± 24.9	< 0.001	135.144
Creatinine (mg/dL)	1.4 ± 1.3	1.4 ± 1.2	1.7 ± 1.4	< 0.001	87.951
Glucose (mg/dL)	159.5 ± 81.9	156.0 ± 81.2	176.2 ± 83.1	< 0.001	116.922
INR	1.6 ± 1.0	1.5 ± 1.0	1.7 ± 1.2	< 0.001	38.95
PT (s)	17.0 ± 11.0	16.8 ± 10.5	18.3 ± 12.7	< 0.001	37.779
PTT (s)	44.9 ± 31.2	44.2 ± 30.6	47.8 ± 33.5	< 0.001	24.801
Myocardial infarct, n (%)				0.569	0.324
Yes	3,005 (22.8)	2,470 (22.7)	535 (23.3)		
No	10,170 (77.2)	8,405 (77.3)	1765 (76.7)		
Congestive heart failure, n (%)				< 0.001	48.181
Yes	4,363 (33.1)	3,459 (31.8)	904 (39.3)		
No	8,812 (66.9)	7,416 (68.2)	1,396 (60.7)		
Chronic pulmonary disease, n (%)				< 0.001	33.154
Yes	3,604 (27.4)	2,863 (26.3)	741 (32.2)		
No	9,571 (72.6)	8,012 (73.7)	1,559 (67.8)		
Peripheral vascular disease, n (%)				< 0.001	28.011
Yes	2011 (15.3)	1,577 (14.5)	434 (18.9)		
No	11,164 (84.7)	9,298 (85.5)	1866 (81.1)		
Diabetes, n (%)				0.055	3.678
Yes	4,250 (32.3)	3,469 (31.9)	781 (34)		
No	8,925 (67.7)	7,406 (68.1)	1,519 (66)		
Renal disease, n (%)				< 0.001	46.63
Yes	3,151 (23.9)	2,474 (22.7)	677 (29.4)		
No	10,024 (76.1)	8,401 (77.3)	1,623 (70.6)		
Liver disease, n (%)				< 0.001	32.622
Yes	886 (6.7)	669 (6.2)	217 (9.4)		
No	12,289 (93.3)	10,206 (93.8)	2083 (90.6)		
Cerebrovascular disease, n (%)				< 0.001	84.545
Yes	2,302 (17.5)	1748 (16.1)	554 (24.1)		
No	10,873 (82.5)	9,127 (83.9)	1746 (75.9)		
Sepsis, n (%)				< 0.001	793.203
Yes	6,223 (47.2)	4,524 (41.6)	1,699 (73.9)		
No	6,952 (52.8)	6,351 (58.4)	601 (26.1)		
Emergency surgery, n (%)				0.305	1.054
Yes	4,194 (31.8)	3,441 (31.6)	753 (32.7)		
No	8,981 (68.2)	7,434 (68.4)	1,547 (67.3)		
Mechanical ventilation, n (%)				< 0.001	498.618
Yes	4,435 (33.7)	3,201 (29.4)	1,234 (53.7)		
No	8,740 (66.3)	7,674 (70.6)	1,066 (46.3)		
Vasopressor, n (%)				< 0.001	68.314
Yes	5,190 (39.4)	4,108 (37.8)	1,082 (47)		
No	7,985 (60.6)	6,767 (62.2)	1,218 (53)		
Sedation, n (%)				< 0.001	258.359
Yes	1998 (15.2)	1,398 (12.9)	600 (26.1)		
No	11,177 (84.8)	9,477 (87.1)	1700 (73.9)		
30-day mortality, n (%)				< 0.001	353.616
No	11,880 (90.2)	10,050 (92.4)	1830 (79.6)		
Yes	1,295 (9.8)	825 (7.6)	470 (20.4)		
Sofa	4.0 (2.0, 6.0)	4.0 (2.0, 6.0)	6.0 (4.0, 10.0)	< 0.001	1100.221
Los hospital	7.1 (4.7, 11.7)	6.5 (4.4, 9.7)	13.8 (8.6, 22.1)	< 0.001	1954.062
Los ICU	2.2 (1.4, 4.0)	2.0 (1.3, 3.1)	5.9 (3.4, 10.6)	< 0.001	2814.961

CVICU, Cardiac Vascular Intensive Care Unit; CCU, Coronary Care Unit; MICU, Medical Intensive Care Unit; MICU/SICU, Medical/Surgical Intensive Care Unit; NICU/NSICU, Neuro/Neuro Surgical Intensive Care Unit; SICU, Surgical Intensive Care Unit; TSICU, Trauma SICU; MBP, mean blood pressure; WBC, white blood cell; BUN, blood urea nitrogen; PT, prothrombin time; PTT, partial thromboplastin time; INR, international normalized ratio; SOFA, Sequential Organ Failure Assessment.

### Nomogram characteristics selection and development

**Figure 2:**
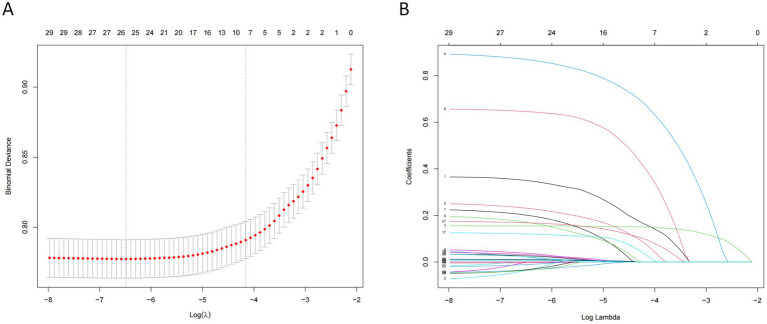
Features selection using the LASSO regression model. **(A)** The largest *λ* value with mean error within single standard deviation is determined through cross-validation. **(B)** Seven coefficients with non-zero values are selected.

**Figure 3:**
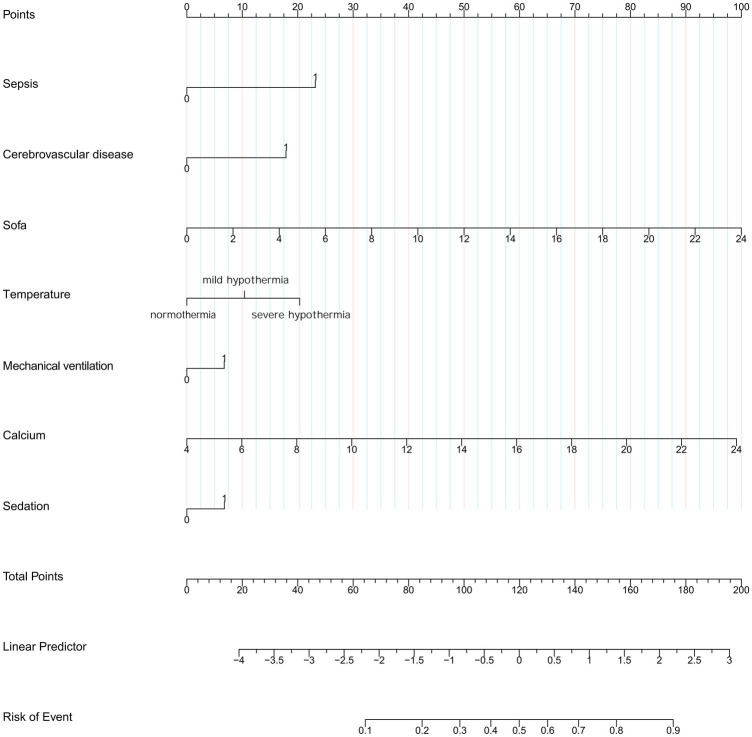
Nomogram to predict the risk to develop delirium in elder admission to ICU. SOFA, Sequential Organ Failure Assessment.

The variables already incorporated into the SOFA scoring system, including MBP, platelet count, creatinine level, and vasopressor use, were not used for LASSO regression, analysis, and cross-verification. Of the 30 variables, we recognized 7 variables as independent delirium predictors (Figure 2). The performance of risk factors was as follows: sepsis (OR: 2.50; 95% CI 2.19–2.86), SOFA score (OR: 1.18; 95%CI 1.16–1.21), sedation (OR: 1.31; 95%CI 1.12–1.52), mechanical ventilation (OR: 1.03; 95% CI 1.17–1.53), severe hypothermia (OR: 1.59; 95% CI 1.41–1.80), calcium levels (OR: 1.22; 95% CI 1.14–1.32), and cerebrovascular disease (OR: 2.03; 95% CI 1.75–2.35) (Table 2). Based on these outcomes, a nomogram was constructed to predict delirium in older patients admitted to the ICU (Figure 3).

**Table 2:** Multivariable logistic regression model for predicting the delirium in elderly patients admitted to ICU.

Variable	Adjusted rate ratio (95% CI)	*p-*value
Mechanical ventilation	1.34 (1.17~1.53)	<0.001
Calcium	1.22 (1.14~1.32)	<0.001
Sepsis	2.5 (2.19~2.86)	<0.001
Sofa	1.18 (1.16~1.21)	<0.001
Sedation	1.31 (1.12~1.52)	0.001
Cerebrovascular disease	2.03 (1.75~2.35)	<0.001
Severe hypothermia	1.59 (1.41~1.8)	<0.001

ICU, intensive care unit.

### Nomogram performance

**Figure 4:**
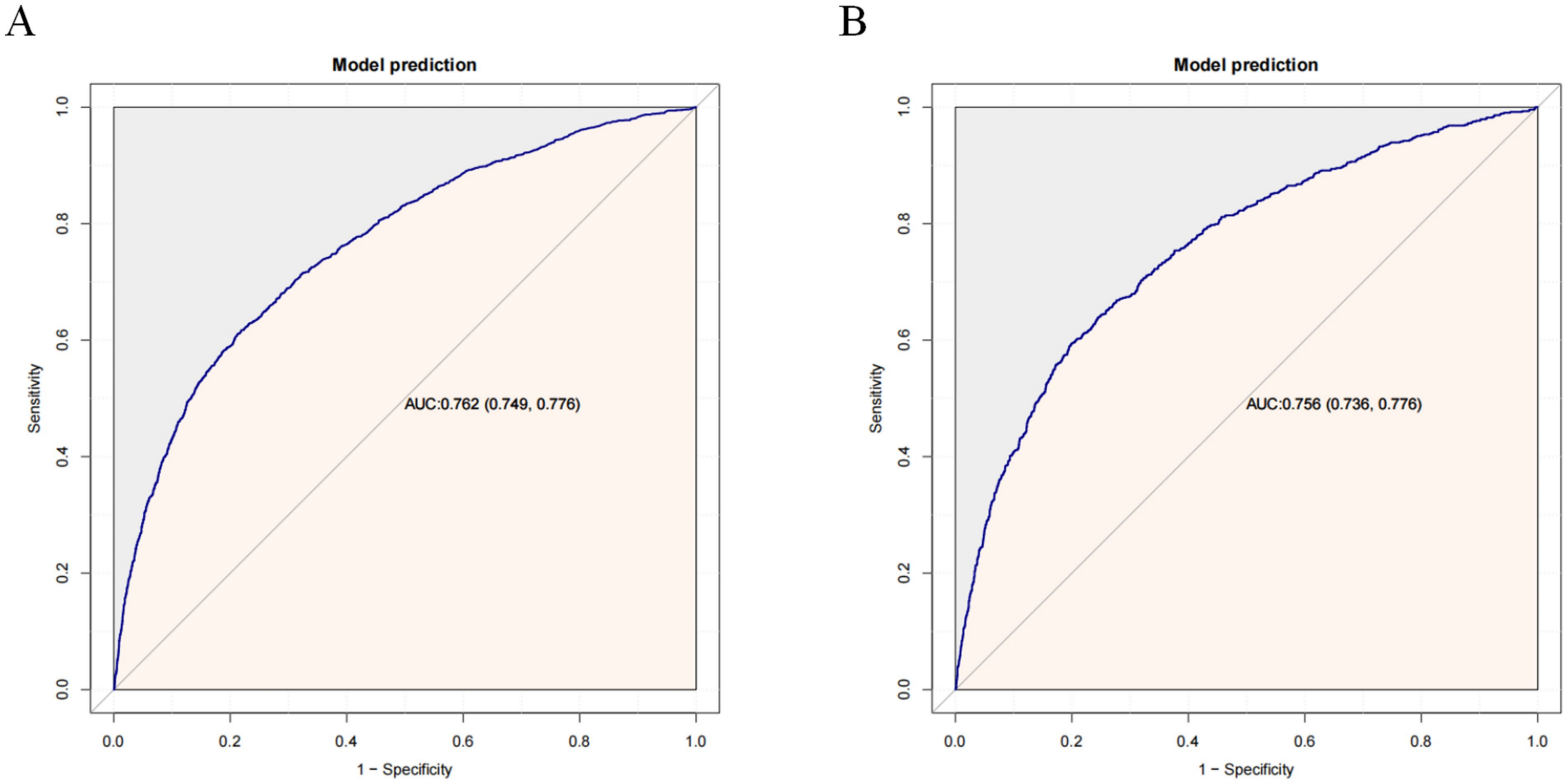
Receiver operating characteristic (ROC) curves in the training set **(A)** and validation set **(B)**. AUC, area under the ROC curve.

**Figure 5:**
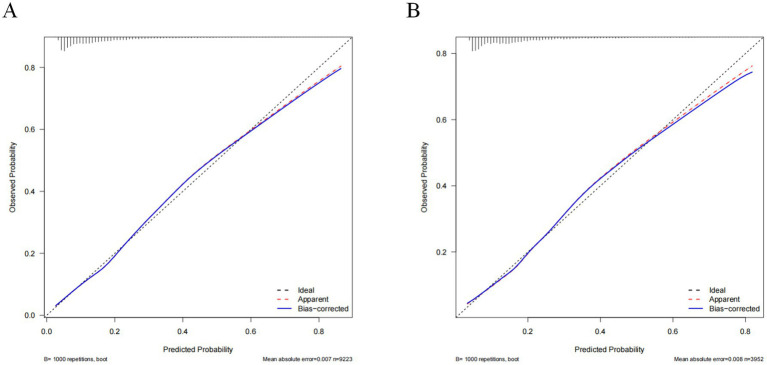
Calibration curves of the nomogram in the training set **(A)** and validation set **(B)**. The horizontal axis depicts the anticipated likelihood of delirium, and the vertical axis illustrates the actual occurrence of diagnosed delirium relative to the total cases. The diagonal dashed line represents the perfect prediction of the ideal model. The red line represents the prediction of the nomogram; the bule line represents the result after bias correction by bootstrapping (1,000 repetitions).

**Figure 6:**
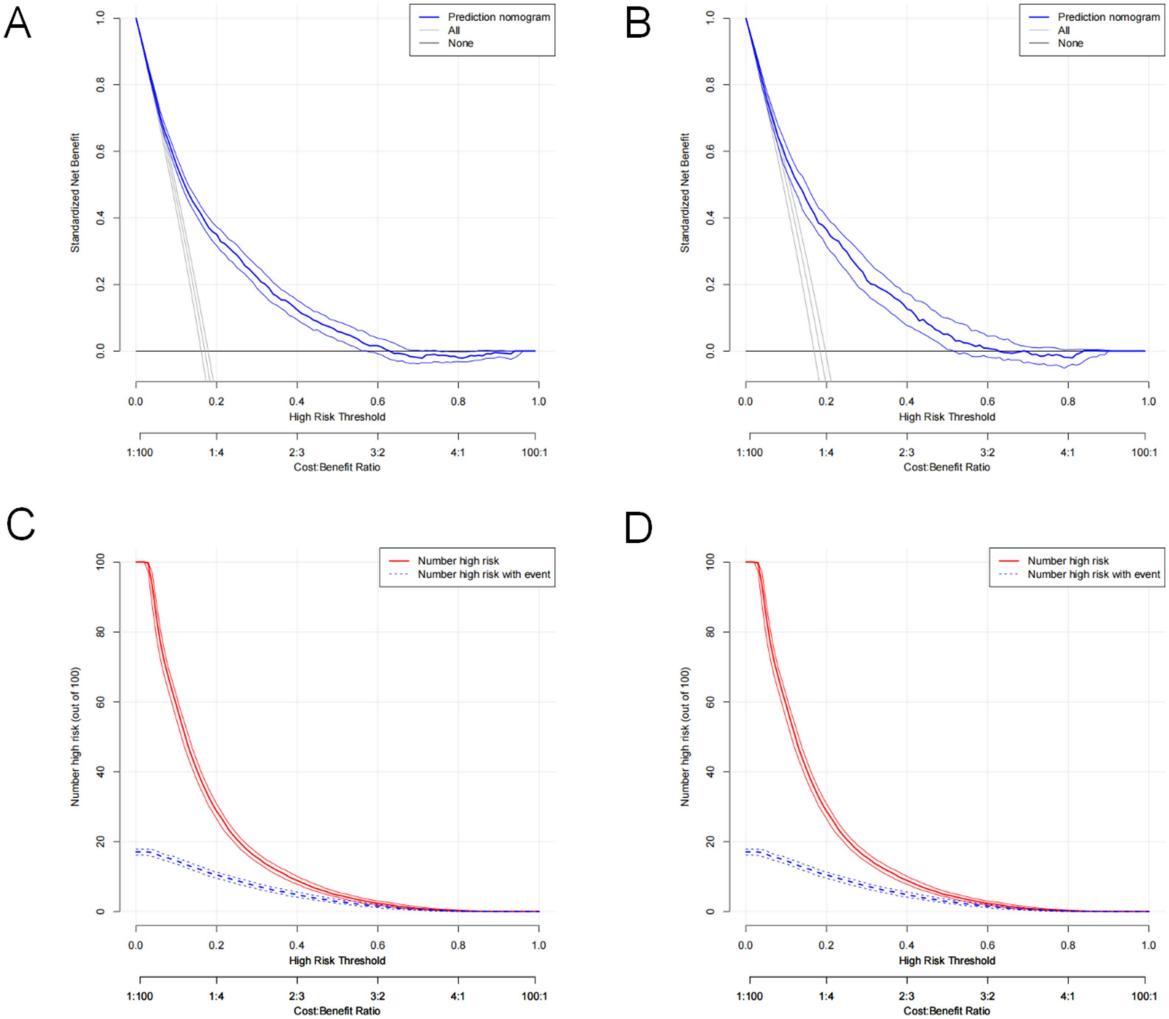
Decision curve analysis of the nomogram in the training set **(A)** and validation set **(C)**. Clinical impact curve analysis of the nomogram in the training set **(B)** and validation set **(D)**.

The constructed model generated an AUC value of 0.762 (95% CI 0.749–0.776) in the training set and 0.756 (95% CI 0.736–0.776) in the verification set (Figure 4). We plotted the calibration curves for the two sets and formed a bias-corrected line using the bootstrap method. Calibration curves were fairly close to the standard diagonal line of 45° (Figure 5), while the Brier score of the nomogram was 0.12 in the training set and 0.128 in verification set, suggesting favorable consistency between the forecast and actual values. Meanwhile, non-significant *p*-values of 0.386 and 0.686 in the two sets were observed in the HL test. Furthermore, the clinical utility of the nomogram was assessed using the CIC and DCA. When the threshold probability was between 0.1 and 0.65, a greater net benefit could be generated from such a forecast model, as indicated by the DCA curve (Figure 6A). The high-risk patient number (number of forecast delirium cases via nomogram) matched well with the high-event-risk patient number (number of true-positive delirium cases), whereas the threshold probability exceeded 0.7, as depicted in the CIC findings (Figure 6B). In the verification set, the DCA and CIC curve also demonstrated the benefit of applying the nomogram in clinical practice (Figures 6C,D). In summary, by combining various evaluation parameters, our model showed an excellent predictive value for delirium in elderly patients.

## Discussion

Here, we established and verified a novel nomogram model for delirium in elderly critically ill patients. We constructed the nomogram using seven variables: sepsis, SOFA score, cerebrovascular disease, sedation, mechanical ventilation, severe hypothermia, and serum calcium levels. We could easily obtain all the predictors in an objective and reliable manner. Our findings implied that this nomogram has good discrimination and satisfactory calibration, indicating potential clinical applicability.

Delirium subtypes were designated as hypoactive, hyperactive, or mixed based on observed behavior. Significantly, hypoactive delirium is often underrated and is associated with a worse prognosis (26). Medical workers, especially in ICUs, are usually drawn to hyperactive and mixed delirium but often overlook hypoactive delirium (27). A delirium forecasting model in daily practice would facilitate clinicians to screen/detect patients who might benefit from delirium prevention. With limited resources, this could reduce waste and misdiagnosis chances effectively, and might accordingly enhance critical patient outcomes. This is an original finding and the clinical significance of our study. Compared with previous delirium prediction models in the ICU setting, our model performed well. A previous study showed that the AUC was significantly lower for early prediction model for delirium in ICU patients (0.68 (95% confidence interval 0.66–0.71)) (28) compared to our model (0.762 (95% confidence interval 0.749–0.776)) in the training set. The DCA curves further illustrated our model’s favorable clinical utility.

Sepsis had the greatest weight in the nomogram, implying that it serves as the most significant predictor and is the strongest predictor of delirium in older patients in our study. Patients with sepsis complicated by delirium are actually not rare. Literature reports that the incidence of sepsis-associated delirium (SAD) varies from 9 to 71% in severe sepsis patients (29). There is still much to learn about the underlying mechanisms of SAD, but currently, it is believed to be an integration of neuroinflammation and disturbances in cerebral perfusion, the blood–brain barrier, and neurotransmitters (30). A systemic inflammatory response to infection causes brain activation, resulting in an appropriate anti-inflammatory response. However, excessive pro-inflammatory mediators entering the brain may impair the blood–brain barrier and cause abnormal cerebral perfusion (31, 32). Various neuroendocrine dysfunctions have been observed in sepsis, including hypothalamic–pituitary–adrenal axis impairment, autonomic dysfunction, and vasopressin deficiency (33, 34). These dysfunctions may alter immunity in a vicious spiral, leading to metabolic derangement and neurological decline (29).

Cerebrovascular disease is a common comorbidity that can lead to delirium in ICU patients. As mentioned previously, cerebrovascular disease is an independent risk factor of ICU-associated delirium (35, 36). Cerebrovascular disease can lead to neurocognitive deficits, which are postulated to have a higher occurrence rate of delirium, likely via altered cerebral networks and a reduced capability to combine sensory inputs (37). Long-term susceptibility to delirium should be considered an integrative aspect of the overall cerebrovascular disease burden (38), which deserves attention.

The SOFA score was originally designed to successively evaluate the severity of organ dysfunction in critical patients with sepsis, which included an assessment of neurological function and the Glasgow Coma score (39). The SOFA scores are widely used in ICUs and are readily available. Previous studies have reported that higher SOFA scores are indicative of delirium (40–42). One delirium forecast model included the SOFA score as one of the six predictors (40), whereas in our forecast model, the SOFA score emerged as one of the most significant predictors.

Temperature fluctuation is a common phenomenon in critical patients that might reflect an inflammatory response (43). Additionally, it may be an indicator of altered hypothalamic thermoregulatory center function in brain injury (44). It is associated with adverse outcomes (45), including an unfavorable decline in neurocognitive function (46). In a recent study of 27,674 patients undergoing major noncardiac surgery, severe hypothermia was found to be a predictor of postoperative delirium (21). However, the correlation between severe hypothermia and delirium in elderly ICU patients should warrant further investigation.

Serum calcium is an important bivalent cation involved in cognitive decline pathophysiology. High serum calcium levels pertain to an exaggerated decline in global cognitive function over the age of 75 years, regardless of sex or education level (47).Serum calcium level is a potential presymptomatic biomarker for cognitive impairment and can predict longitudinal cognitive decline and conversion (48). In our study, serum calcium levels were correlated with delirium development. Our results were consistent with those of previous studies.

Mechanical ventilation and sedation are critical interventions in the ICU, particularly in patients requiring prolonged mechanical ventilation. Previous studies have revealed that delirium occurred in 38 to 80% of patients who underwent mechanical ventilation in the ICU (49, 50). Mesa et al. demonstrated that a mechanical ventilation duration of 7 days or longer markedly increases the risk of both delirium and mortality (51). Mechanical ventilation appears to be a risk factor for delirium as it could contribute to immobilization, inflammation, physiological stress, sedation, and sleep rhythm disturbance (52). Furthermore, sedation is considered a significant risk factor for delirium, particularly in elderly patients (53). It is crucial to determine optimal sedation strategies and minimize ventilation time to prevent delirium.

Our study had some limitations. First, this was a single-center retrospective study spanning 2009–2018; therefore, a potential selection bias was inevitable. Second, due to database limitations, we missed certain social attributes of older patients, such as income and subjective social status. Third, because > 20% of the data in the dataset were missing, we lacked some key clinical parameters. Fourth, this was a large-sample study, and the robustness of the model was confirmed through internal validation. However, it is crucial to validate its effectiveness in different medical institutions and ensure its applicability in broader scenarios.

## Conclusion

In such a large cohort of critically ill patients, we provided a visual and personalized nomogram that enables clinicians to identify and detect delirium in older patients in a timely manner. By precisely assessing an individual’s risk of delirium, physicians can implement effective interventions to decrease the incidence of delirium and thus improve prognosis.

## Data Availability

The original contributions presented in the study are included in the article/Supplementary material, further inquiries can be directed to the corresponding author.

## Footnotes

^1^ http://www.R-project.org
