# Supplementary material for: Development and internal verification of nomogram for forecasting delirium in the elderly admitted to intensive care units: an analysis of MIMIC-IV database
Source: Front Neurol. 2025 May 13;16:1580125. doi: 10.3389/fneur.2025.1580125 (PMC12106030; doi:10.3389/fneur.2025.1580125)
Supplement: Supplementary file 1 [file Table_1.docx]

| Variables | Missing amount |
| --- | --- |
| Creatinine | 53 |
| Chloride | 68 |
| BUN | 53 |
| calcium | 1438 |
| WBC | 63 |
| platelets | 62 |
| hemoglobin | 63 |
| glucose | 55 |
| Temperature | 541 |
| Rest rates | 14 |
| MBP | 2 |
| bicarbonate | 67 |
| PTT | 935 |
| PT | 872 |
| INR | 872 |

Tabel S1 Missing data in detail. Abbreviations: BUN, blood urea nitrogen; WBC, white blood cell; MBP, mean blood pressure; PT, prothrombin time; PTT, partial thromboplastin time; INR, international normalized ratio.
